# Supplementary material for: Natural variation of YELLOW SEEDLING1 affects photosynthetic acclimation of Arabidopsis thaliana
Source: Nat Commun. 2017 Nov 10;8:1421. doi: 10.1038/s41467-017-01576-3 (PMC5680337; doi:10.1038/s41467-017-01576-3)
Supplement: Supplementary file 1 — Supplementary Info [file 41467_2017_1576_MOESM1_ESM.doc]

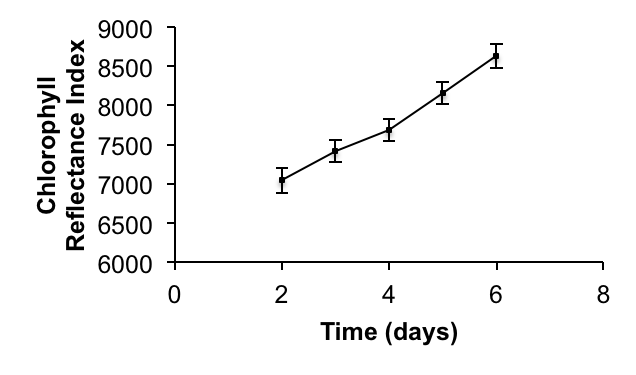


**AD**


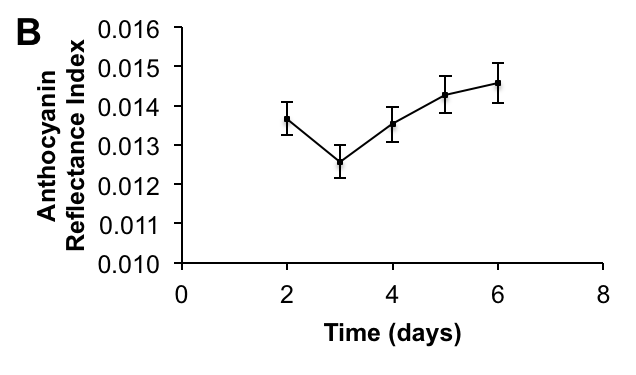


**Supplementary Figure 1. Chlorophyll and anthocyanin reflectance indices**

**(a)** Chlorophyll reflectance index of rosettes of Col-0 (± standard error of the mean (s.e.m.); N=3). **(b)** Anthocyanin reflectance index of rosettes of Col-0 (±s.e.m.; N=3). Both the chlorophyll reflectance indices and the anthocyanin reflectance indices were determined once a day (day 3 is the first day of high irradiance).

**
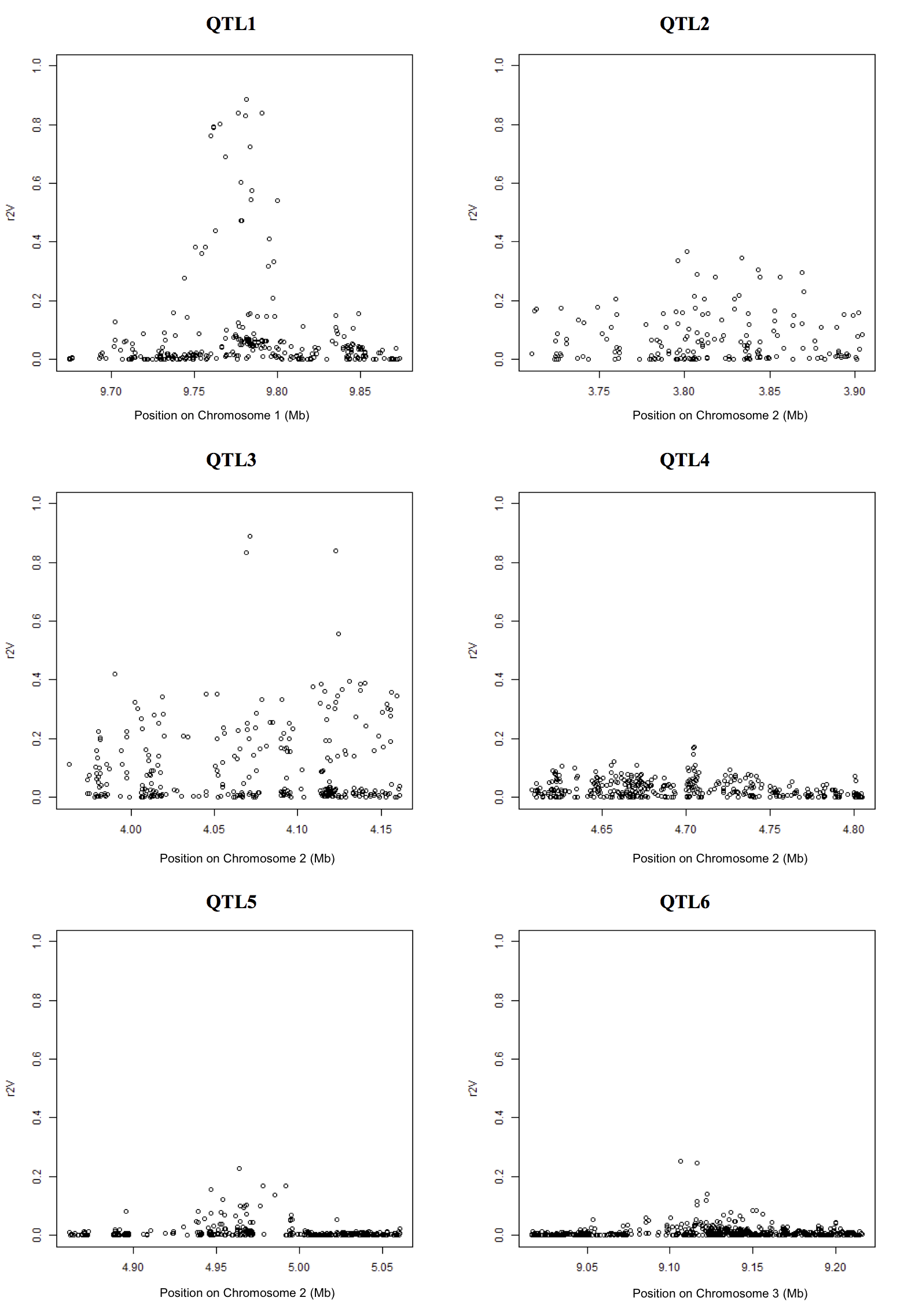
**

**
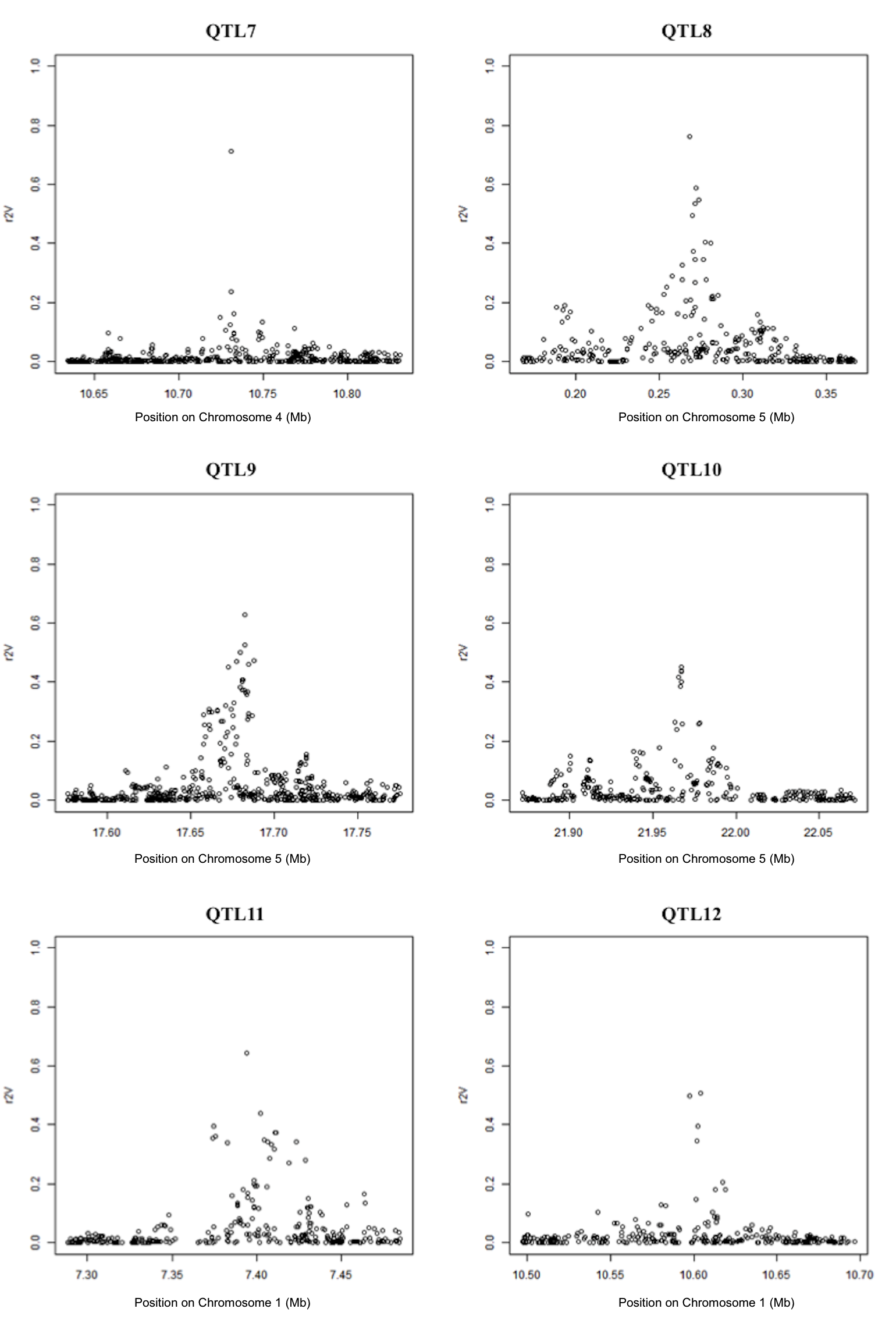
**

**
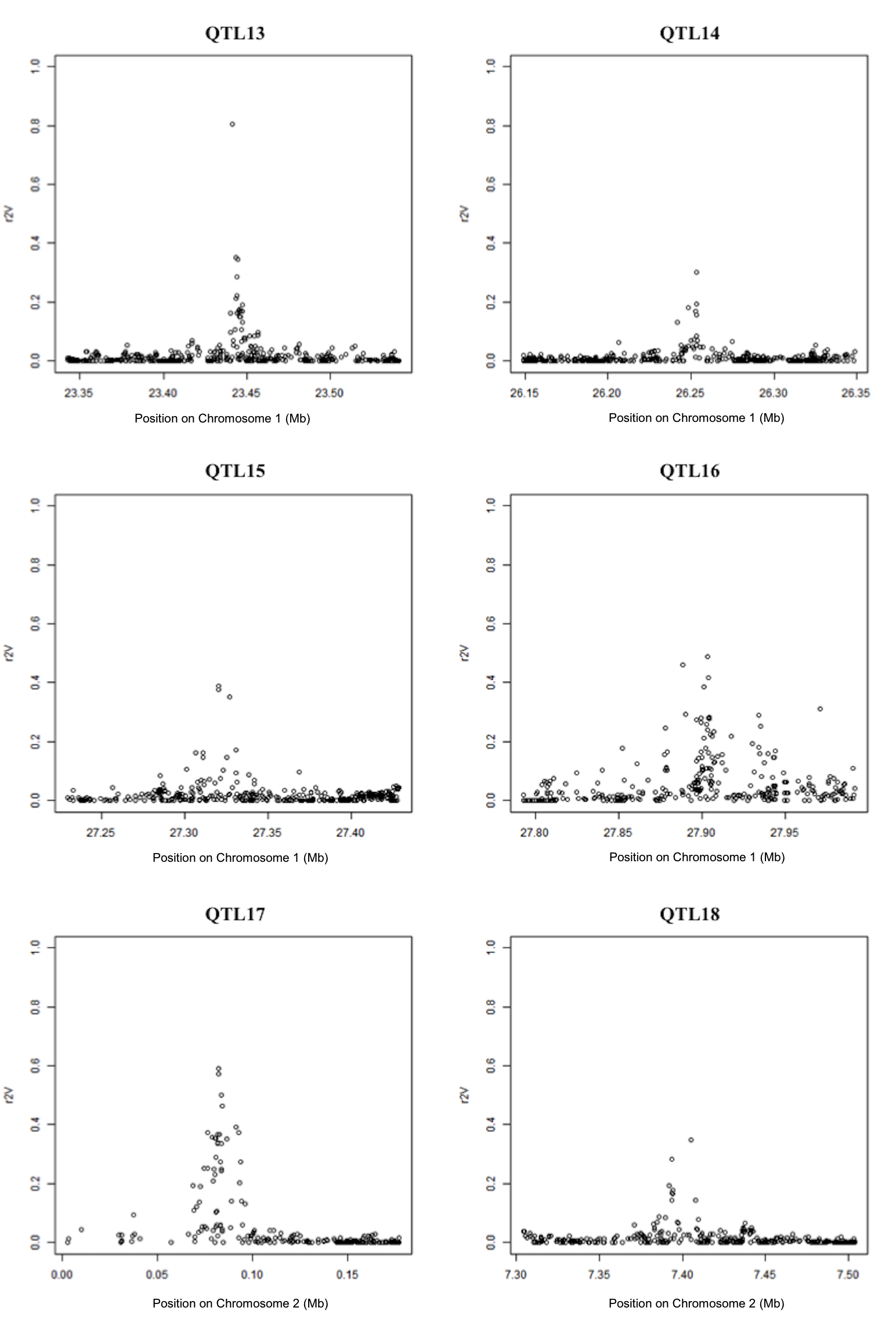
**

**
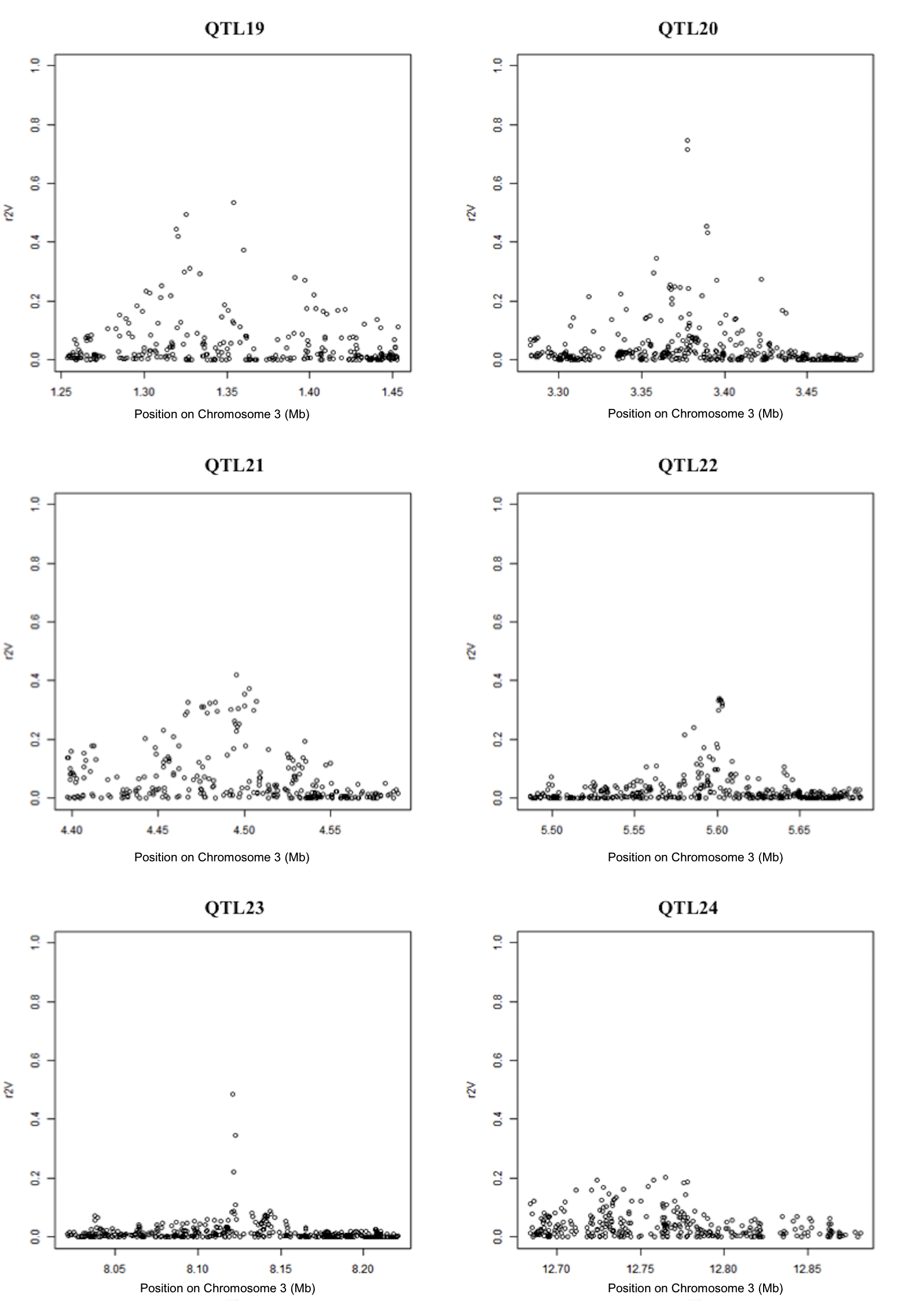
**

**
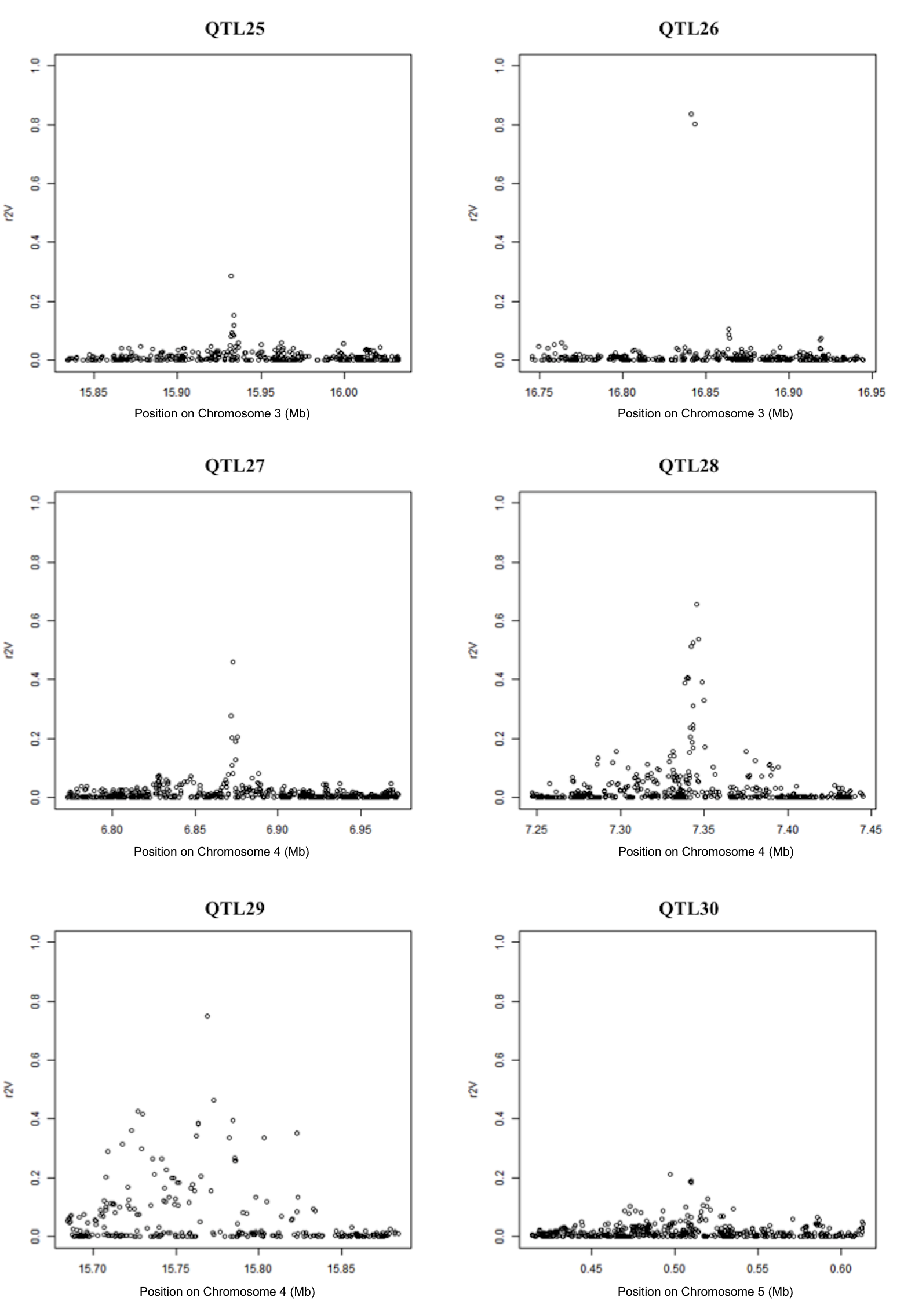
**

**
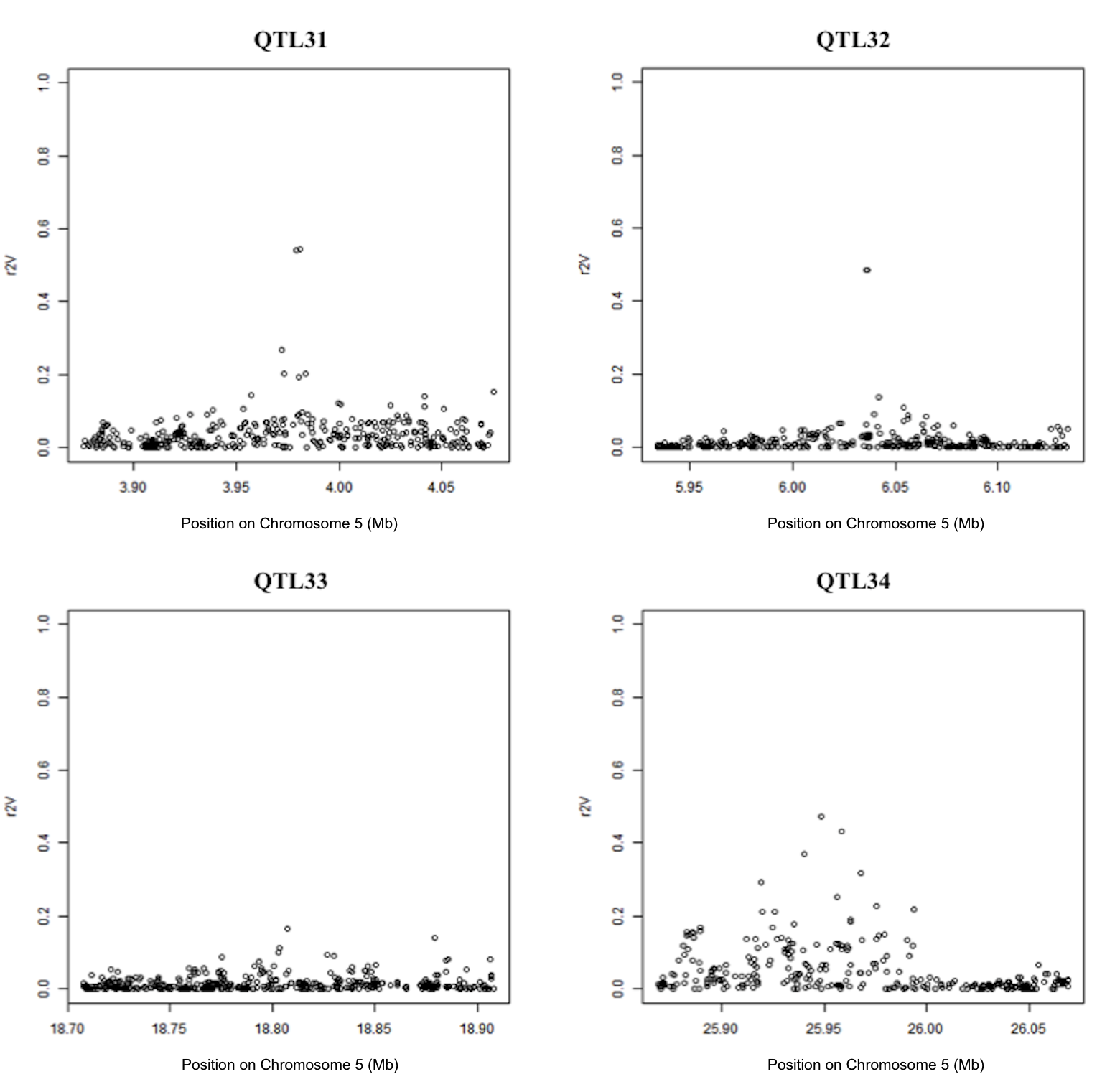
**

**Supplementary Figure 2. Linkage disequilibrium (LD).**

Local LD (r2v) along the chromosomal region (Mb) of the 34 QTLs presented in Figure 2.

**A**

**
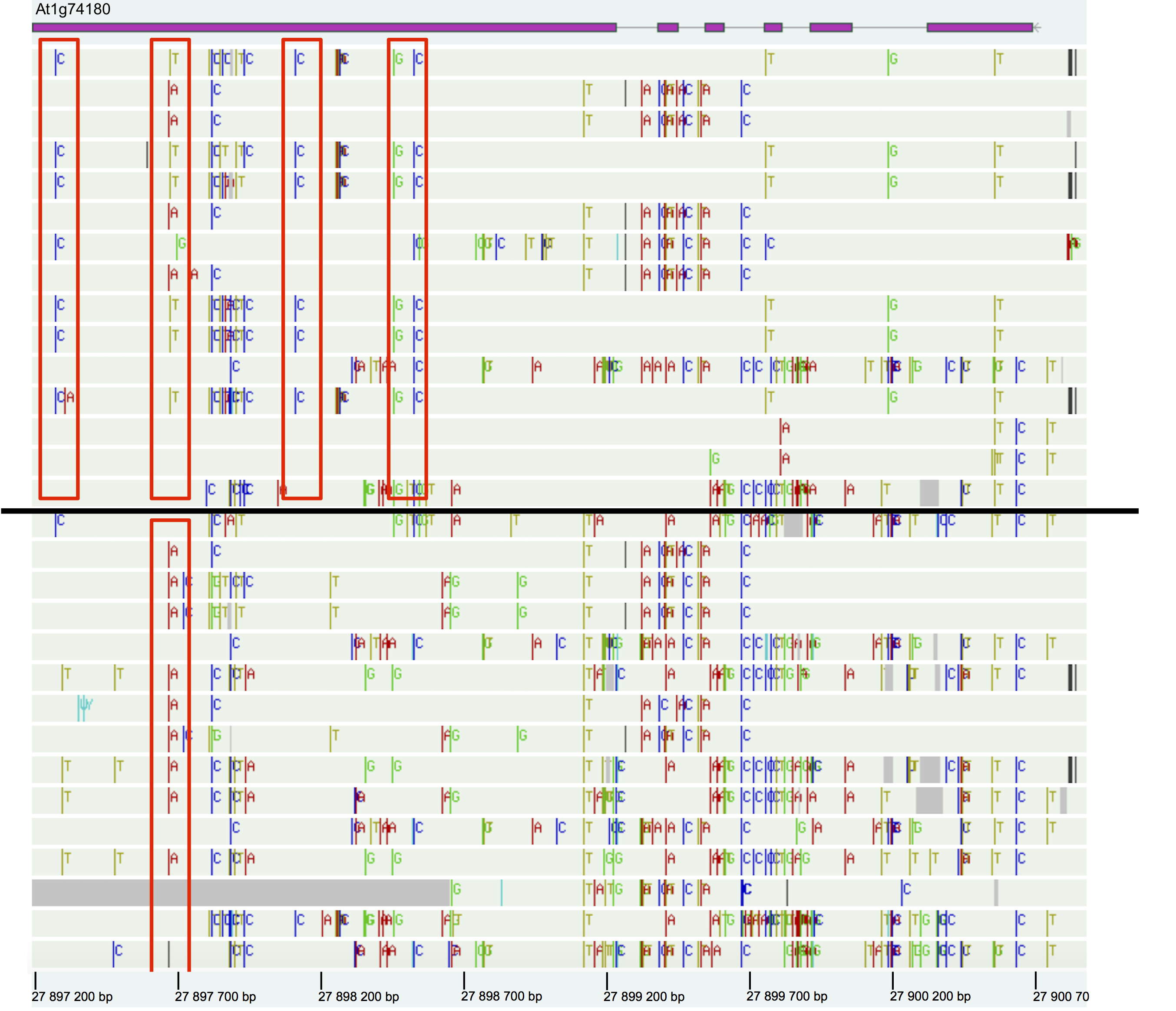
**

**B**

**
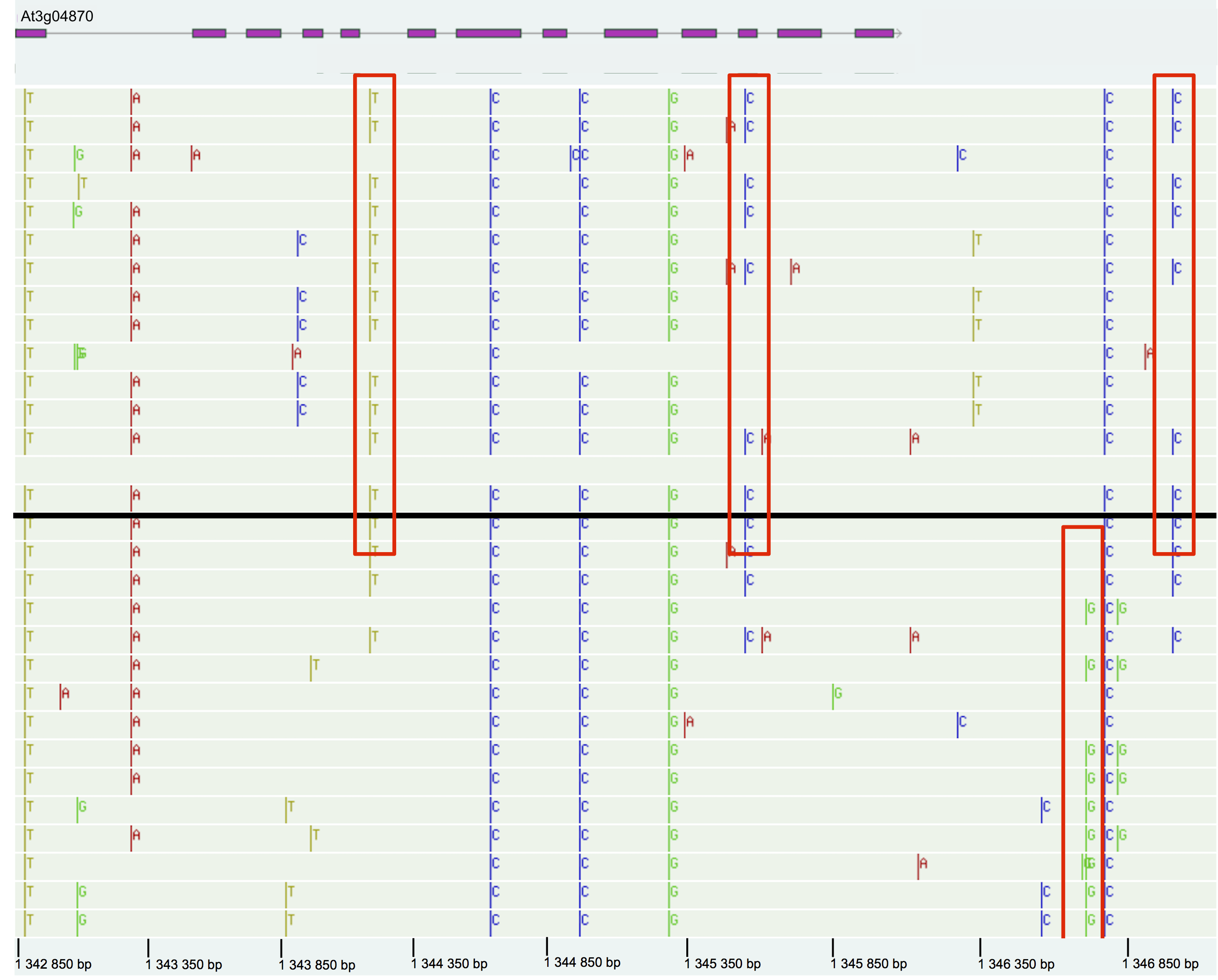
**

**C**

**
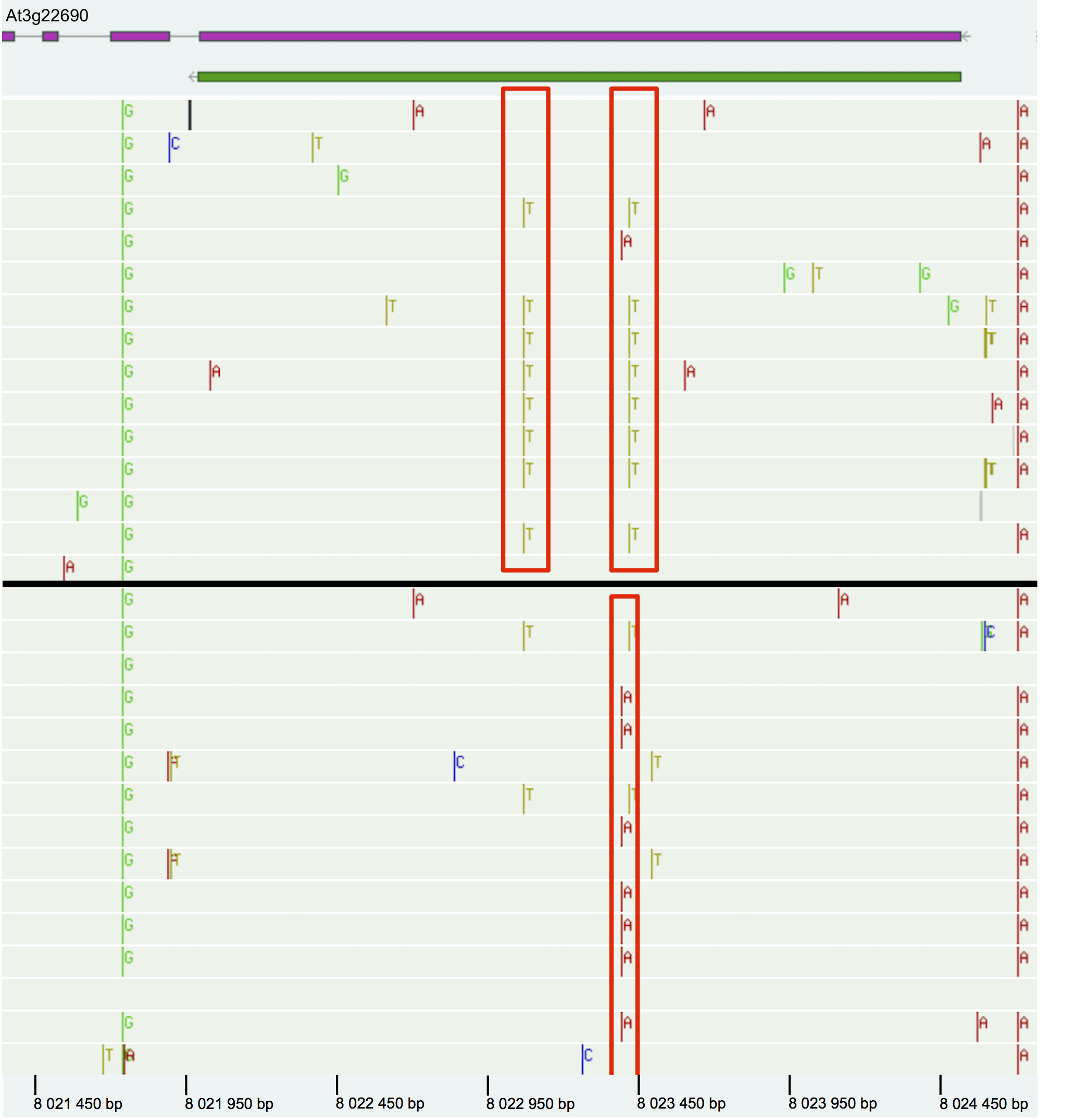
**

**D**

**
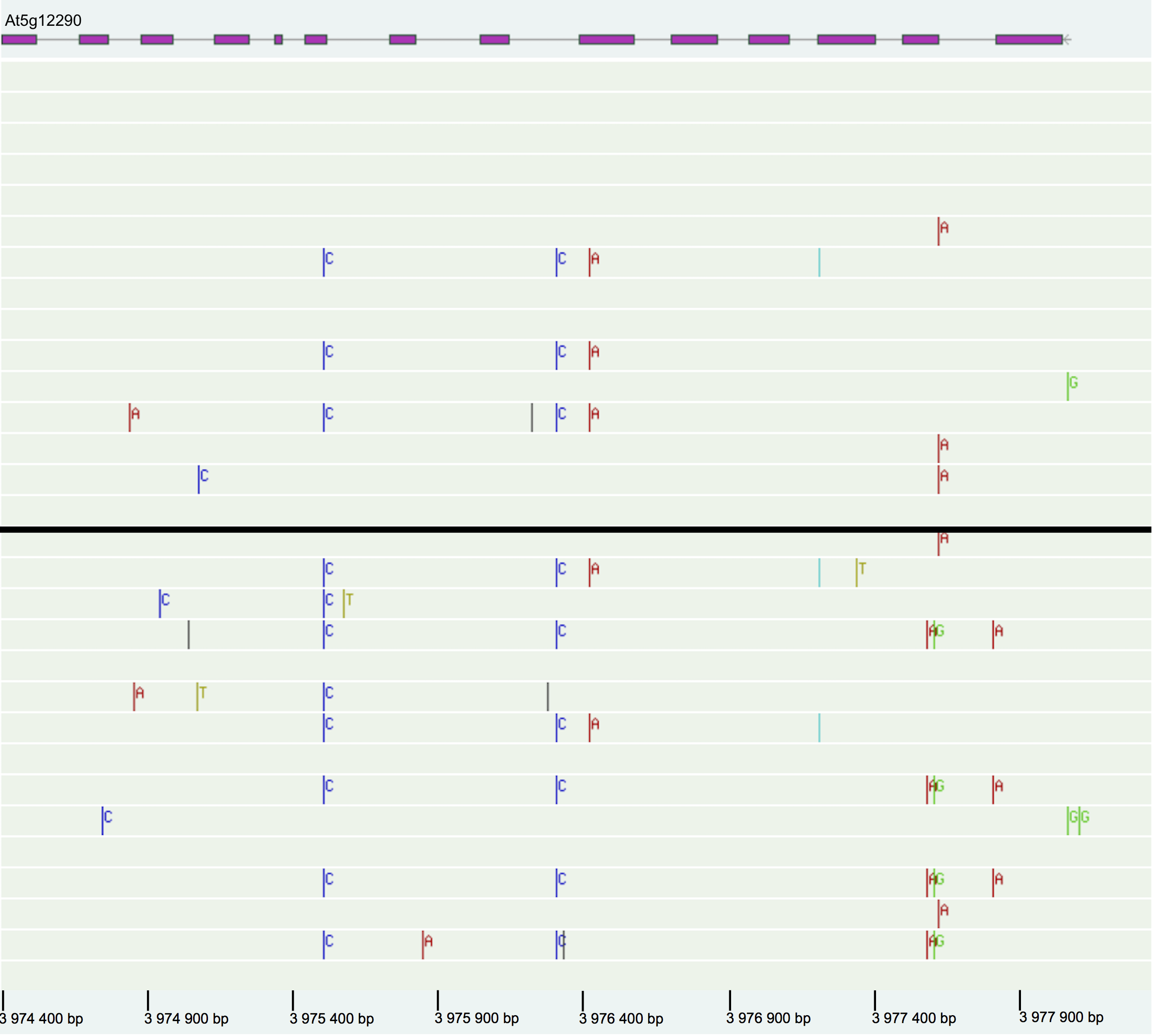
**

**E**

**
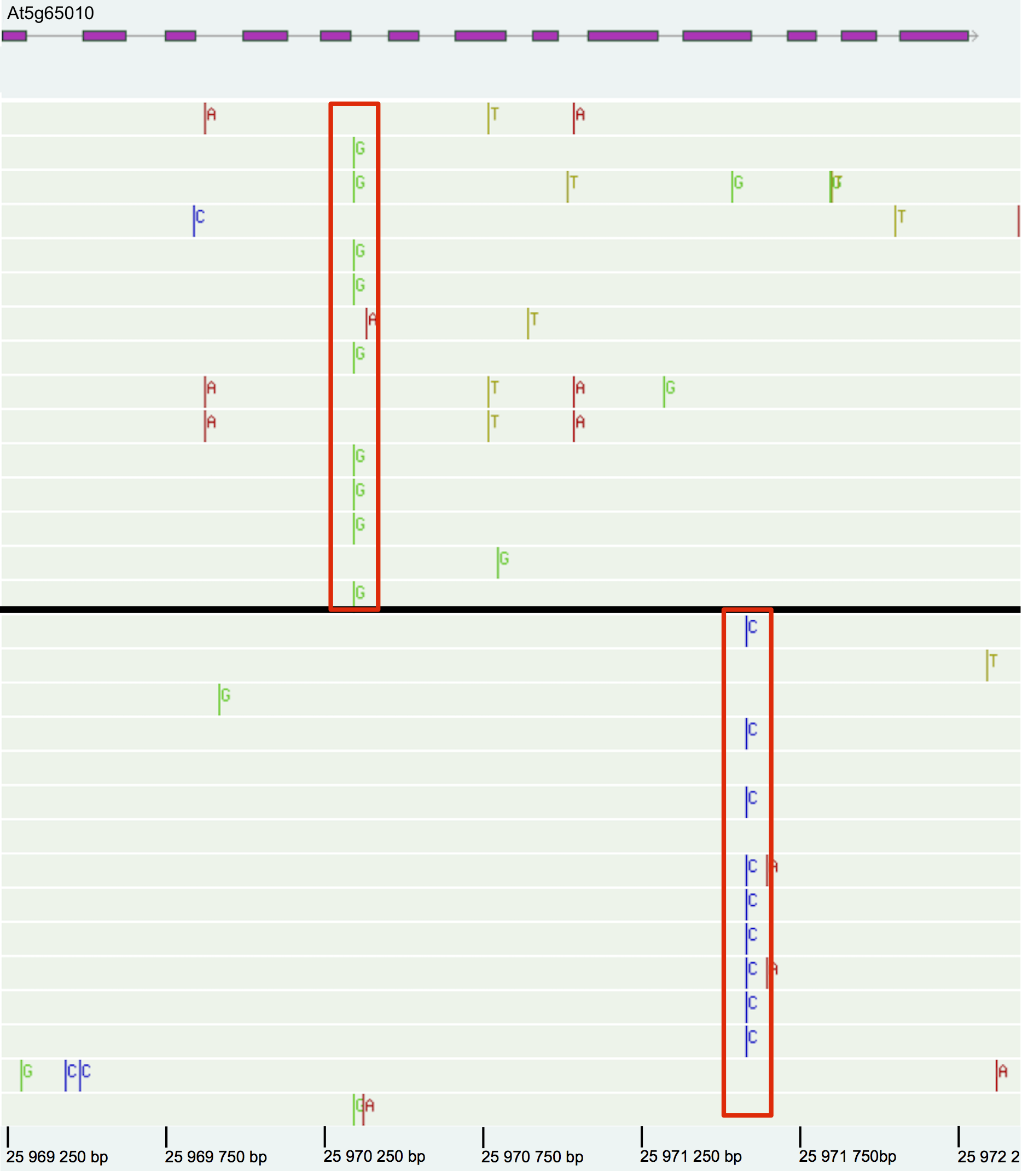
**

**Supplementary Figure 3. Haplotype analysis of candidate genes**

Haplotype analysis of candidate genes associated with the strongest QTLs. specific for HL irradiance. and with the most promising polymorphisms segregating when comparing accessions with extreme phenotypes. These include 15 accessions with the highest photosynthesis efficiency. measured 1 hour after the irradiance increase (above the black line; representing the accessions CS28786. CS28787. CS76128. CS76171. CS28054. CS76133. CS76296. CS76099. CS76239. CS76232. CS76251. CS76222. CS76231. CS28780. and CS28729) and 15 accessions with the lowest photosynthesis efficiency 1 hour after the irradiance increase (beneath the black line. representing the accessions CS76214. CS28369. CS76166. CS76097. CS28685. CS28640. CS76098. CS76139. CS76268. CS76087. CS76198. CS76172. CS76193. CS76109. and CS76196) for **(A)** At1g74180; **(B)** At3g04870 and At3g04880; **(C)** Ag3g22690; **(D)** At5g12290; and **(E)** At5g65010. Each panel represents 5 Kb, chromosome positions are indicated at the bottom of each panel. SNPs differing from the Col-0 reference genome sequence (not indicated) are marked with a letter. SNPs distinguishing contrasting phenotypes are boxed in red. Images are obtained from http://signal.salk.edu/atg1001/3.0/gebrowser.php.

**
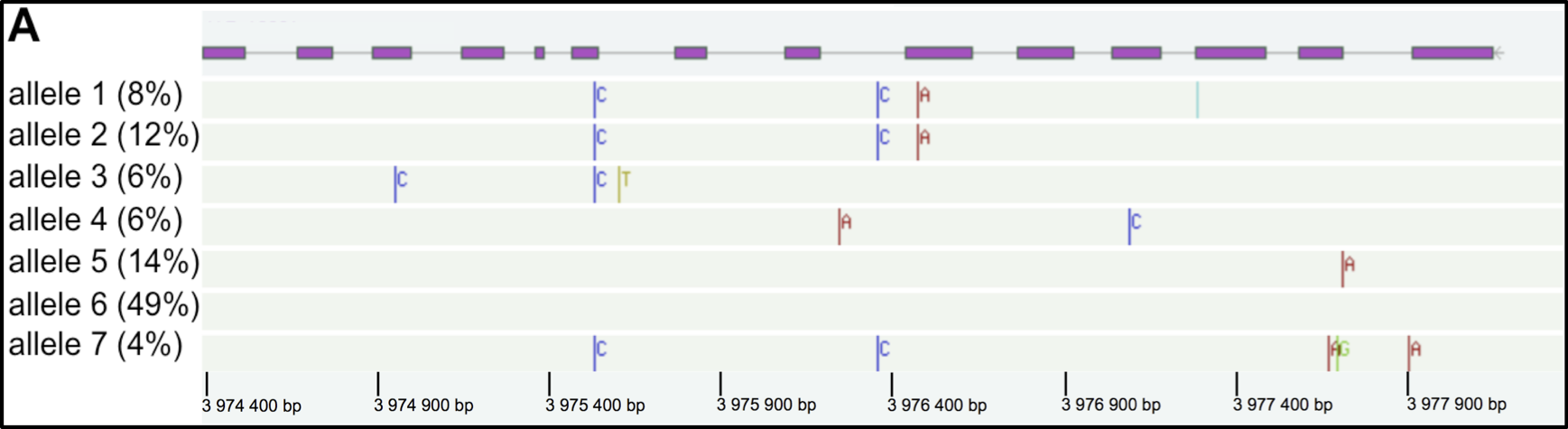
**

**
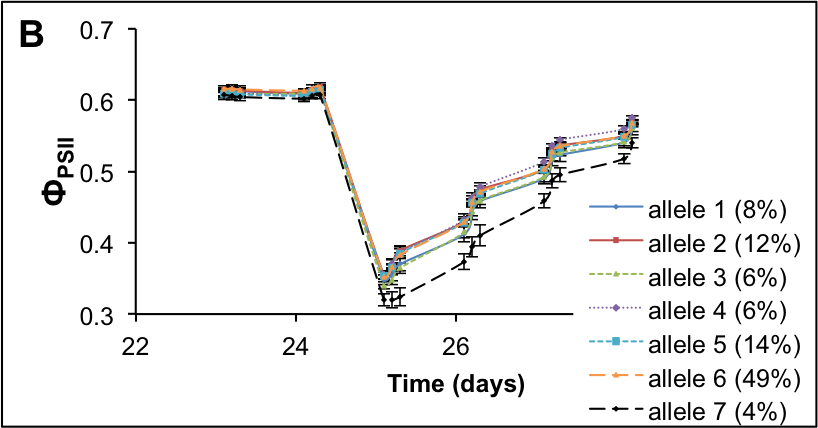
**

**
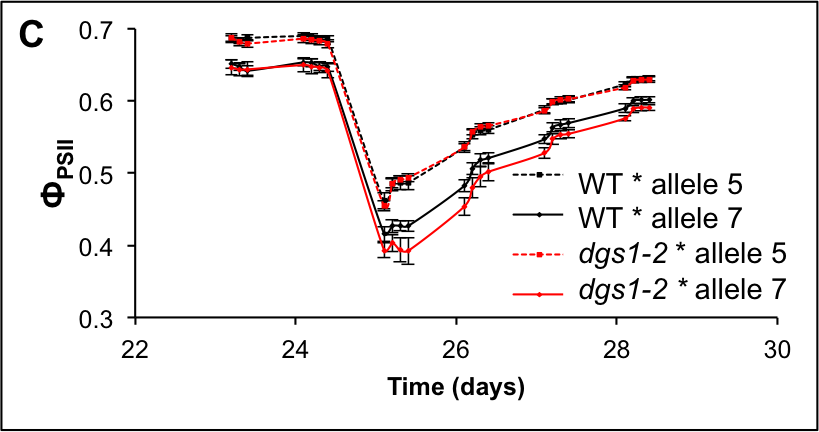
**

**Supplementary Figure 4. Characterization of natural alleles of *DIGALACTOSYL DIACYLGLYCEROL DEFICIENT 1 (DGD1) SUPPRESSOR 1 (DGS1)***

**(A)** Overview of haplotype alleles and frequencies for the *DGS1* gene (At5g12290). he gene orientation is 3’ to 5’. SNPs differing from the Col-0 reference genome sequence (not indicated) are marked. Only haplotypes with allele frequency ≥ 4% are shown, chromosome positions are indicated at the bottom. The image is obtained from http://signal.salk.edu/atg1001/3.0/gebrowser.php; **(B)** average photosynthesis efficiencies (Ф_PSII_) (±s.e.m.; N is variable. depending on the haplotype group size) of the seven haplotype alleles before and after an increase in radiance at the onset of day 25. Only allele 7 responds statistically significantly different from the other alleles; **(C)** In a quantitative complementation analysis. alleles 5 and 7 respond similarly regarding Ф_PSII_ in F1 plants (average Ф_PSII_ ±s.e.m.; N=16) upon crossing appropriate accessions with the Col wild type (WT) or with the T-DNA insertion mutant line SAIL_391_F04 (*dgs1-2*).

**
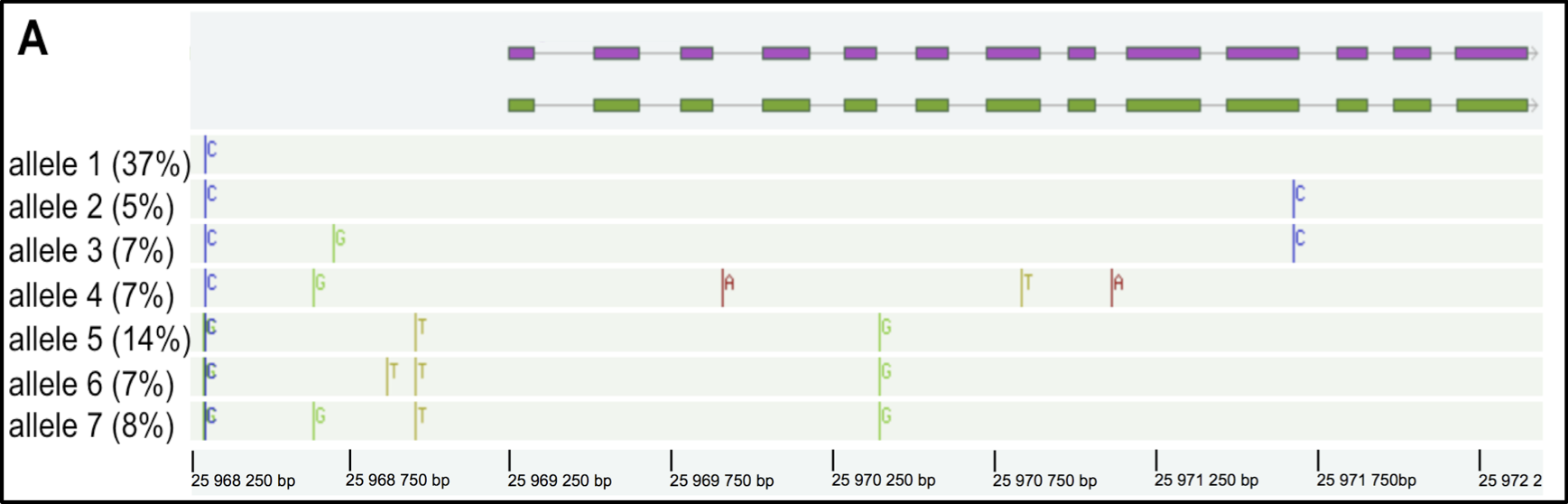
**

**
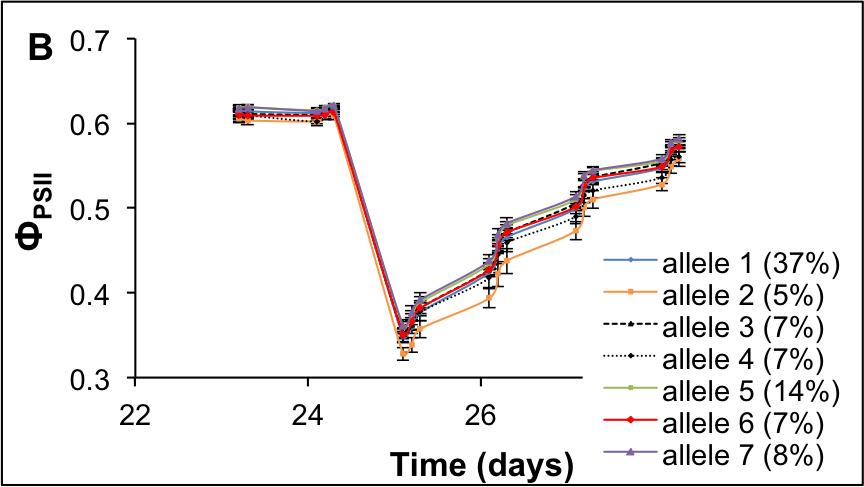
**

**
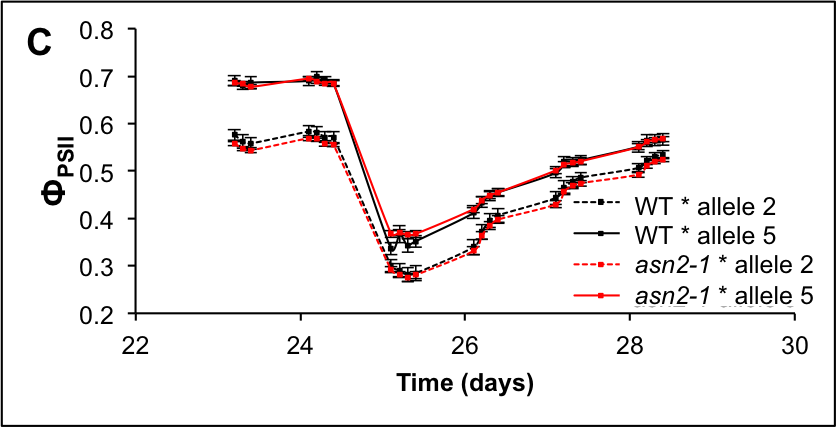
**

**Supplementary Figure 5. Characterization of natural alleles of *ASPARAGINE SYNTHETHASE 2 (ASN2)***

**(A)** Overview of haplotype alleles and frequencies for the *ASN2* gene (At5g65010). he gene orientation is 5’ to 3’. SNPs differing from the Col-0 reference genome sequence (not indicated) are marked. Only haplotypes with allele frequency ≥ 4% are shown, chromosome positions are indicated at the bottom. The image is obtained from http://signal.salk.edu/atg1001/3.0/gebrowser.php; **(B)** average photosynthesis efficiencies (Ф_PSII_) (±s.e.m.; N is variable. depending on the haplotype group size)) of the seven haplotype alleles before and after an increase in radiance at the onset of day 25. Only allele 2 responds statistically significantly different from the other alleles; **(C)** In a quantitative complementation analysis. alleles 2 and 5 respond similarly regarding Ф_PSII_ in F1 plants (average Ф_PSII_ ±s.e.m.; N=16) upon crossing appropriate accessions with the Col wild type (WT) or with the T-DNA insertion mutant line SALK_043167 (*asn2-1*).


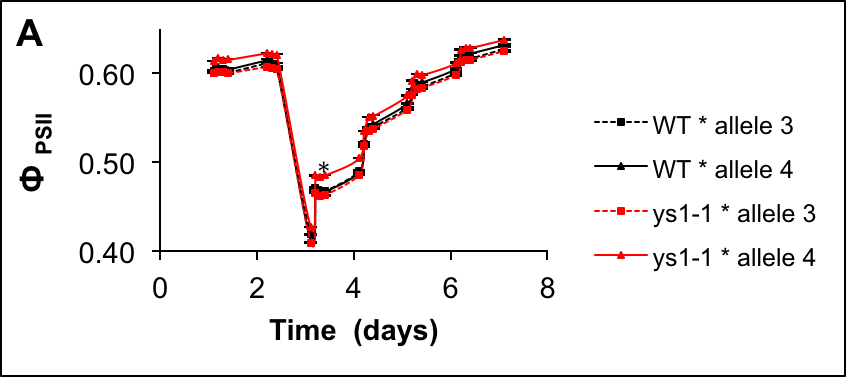


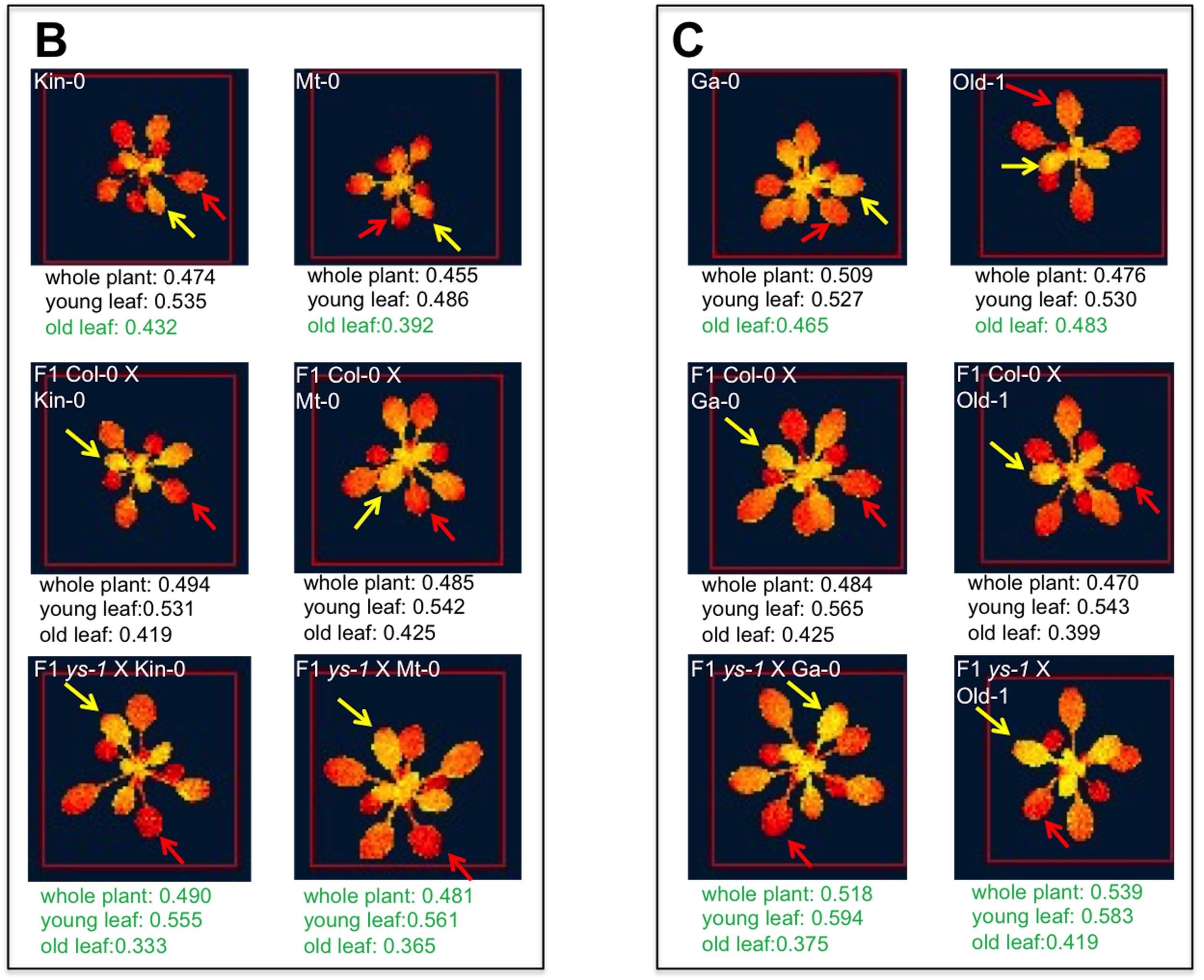


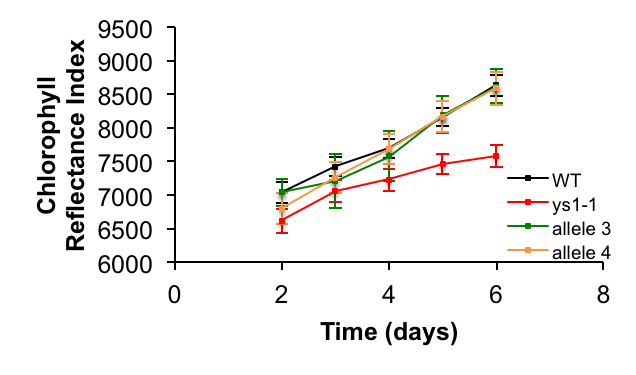


**D**


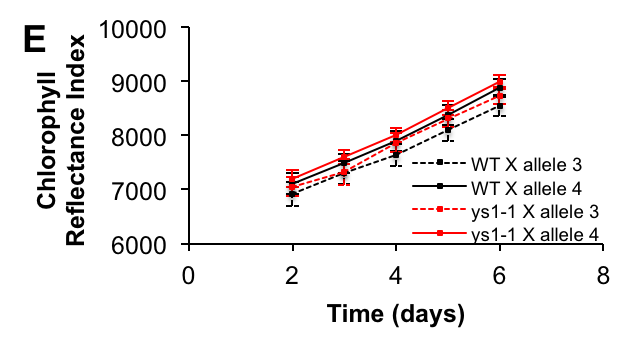


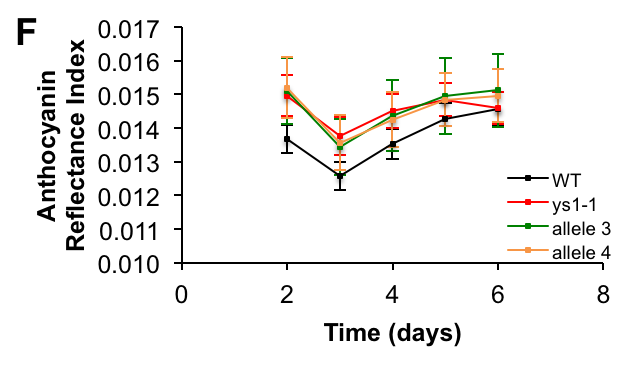


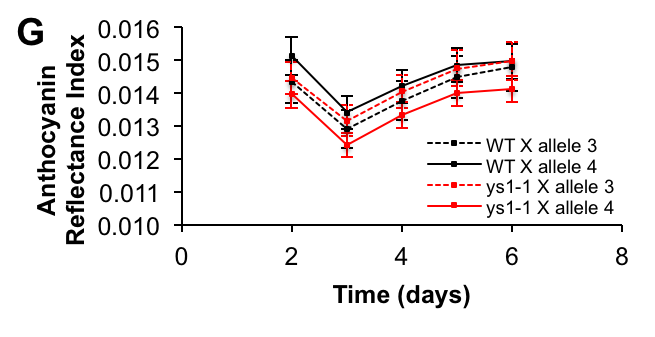


**Supplementary Figure 6. Quantitative complementation of *YS1*. additional data**

**(A)** The quantitative complementation experiment (Fig 4c) was repeated for final confirmation based on new crosses of Col-0 and the *ys1-1* mutant to three different accessions homozygous for the *YS1-3* allele (“allele 3”; Kin-0 (CS76153). Mt-0 (CS76192). and Ren-1 (CS76218)). and comparing it to new crosses of Col-0 and the *ys1-1* mutant to two different accessions homozygous for the *YS1-4* allele (“allele 4”; Old-1 (CS28583) and Ga-0 (CS76133)) in a separate experiment with identical experimental settings. The average Ф_PSII_ (±s.e.m.. N=16) of these F1 progenies is shown. confirming natural genetic variation at *YS1* affecting Ф_PSII_ acclimation in response to an irradiance increase.

* indicates a significant allelic effect (p<0.05; T-test) on the Ф_PSII_ difference at the indicated time point between the different *YS1-1* (WT) x accession and *ys1-1* x accession F1 progenies.

**(B + C)** Chlorophyll fluorescence Ф_PSII_ image analysis of whole plants. young leaves (yellow arrow). or old leaves (red arrow). of two accessions carrying the *YS1-3* allele (Kin-0 and Mt-0) and two accessions carrying the *YS1-4* allele (Ga-0 and Old-1). The mean Ф_PSII_ values are indicated below the false colour images (N= 21 plants) measured respectively for the whole plant. one young leaf. or one old leaf. Values in green are significantly different between panels B and C. Representative false colour images are shown. taking a plant with the average for whole plant closest to the median whole-plant Ф_PSII_ value for each genotype.

**(D+E)** Chlorophyll reflectance index of rosettes of (**D**) Col-0 (WT. *YS1-1*). the *ys1-1* mutant and accessions carrying the *YS1-3* allele or the *YS1-4* allele (±s.e.m; N=4); and of (**E**) F1 quantitative complementation progeny of Col-0 (WT. *YS1-1*) and the *ys1-1* mutant. crossed to either three different accessions homozygous for the *YS1-3* allele. or two different accessions homozygous for the *YS1-4* allele (±s.e.m; N=16). The chlorophyll reflectance indices were determined once a day in response to increased irradiance (day 3 in the figure corresponds to day 25 in the experiment. which is the first day of high irradiance).

**(F+G)** Anthocyanin reflectance index of rosettes of (**D**) Col-0 (WT. *YS1-1*). the *ys1-1* mutant and accessions carrying the *YS1-3* allele or the *YS1-4* allele (±s.e.m; N=4); and of (**E**) F1 quantitative complementation progeny of Col-0 (WT. *YS1-1*) and the *ys1-1* mutant. crossed to either three different accessions homozygous for the *YS1-3* allele. or two different accessions homozygous for the *YS1-4* allele (±s.e.m; N=16). The anthocyanin reflectance indices were determined once a day in response to increased irradiance (day 3 in the figure corresponds to day 25 in the experiment. which is the first day of high irradiance).


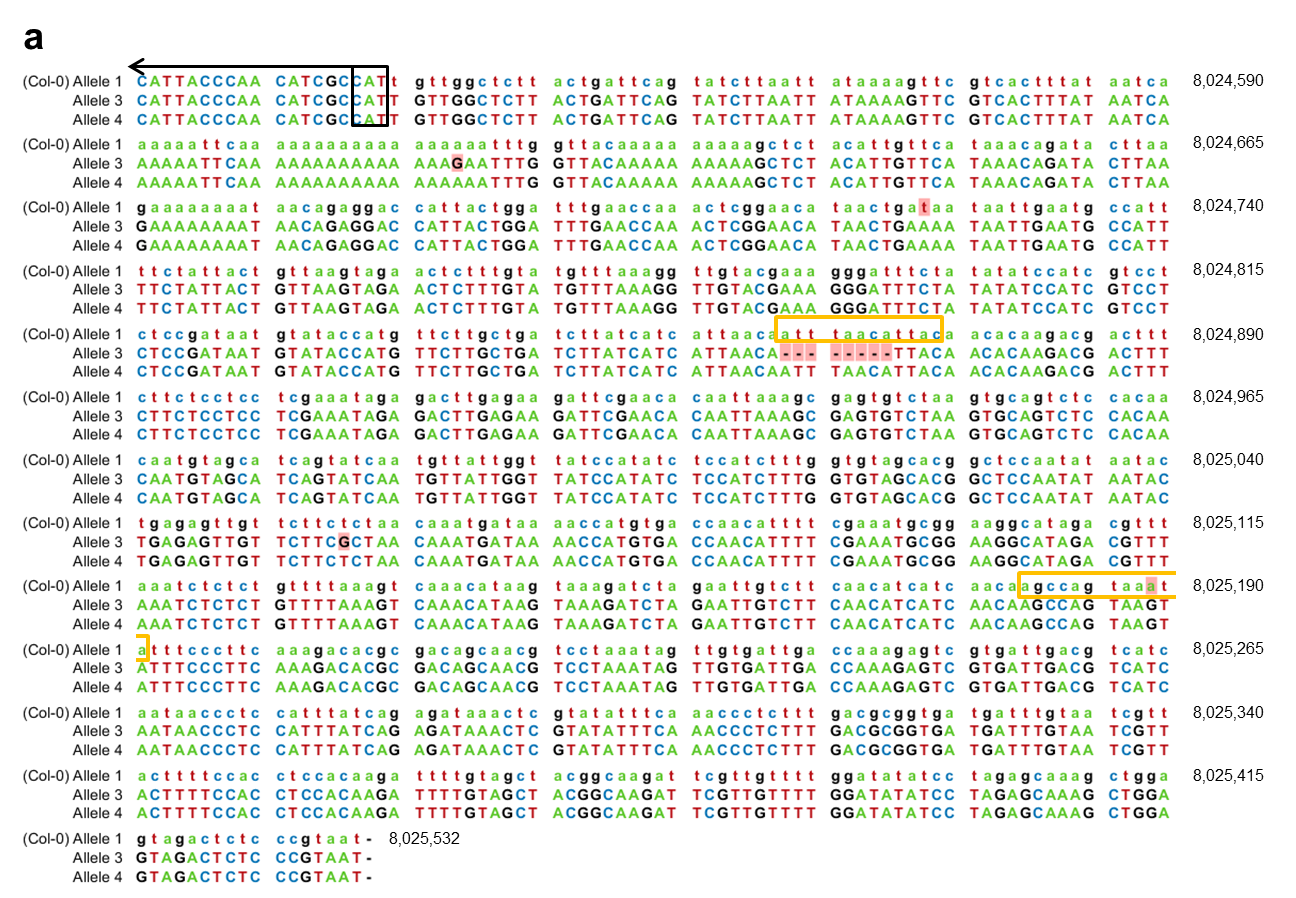


**Supplementary Figure 7. Genomic DNA sequence of the promoters of *YS1* alleles 1. 3 and 4.**

*YS1* promoter sequence alignment (NB. sequence is indicated 3’ to 5’. as *YS1* is positioned in reverse orientation on chromosome 3 between 8021229 - 8024534 bp). The reverse complementary of the *YS1* ATG-start codon is indicated with an arrowed box. with the arrow indicating the direction of translation. Polymorphisms distinguishing the alleles are highlighted in pink. The yellow boxes indicated potential GT-1 binding sites (in opposite orientations relative to each other).

**
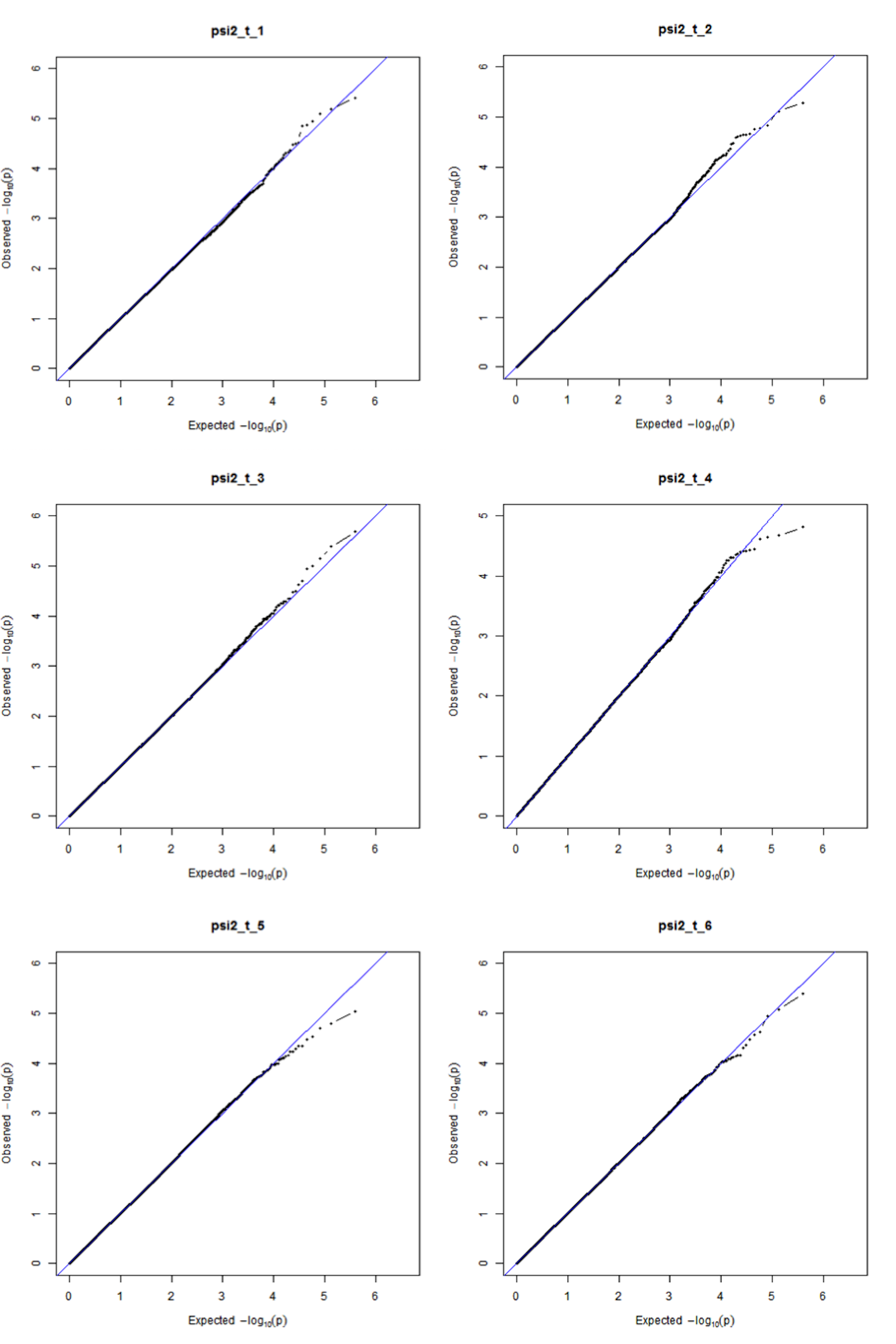

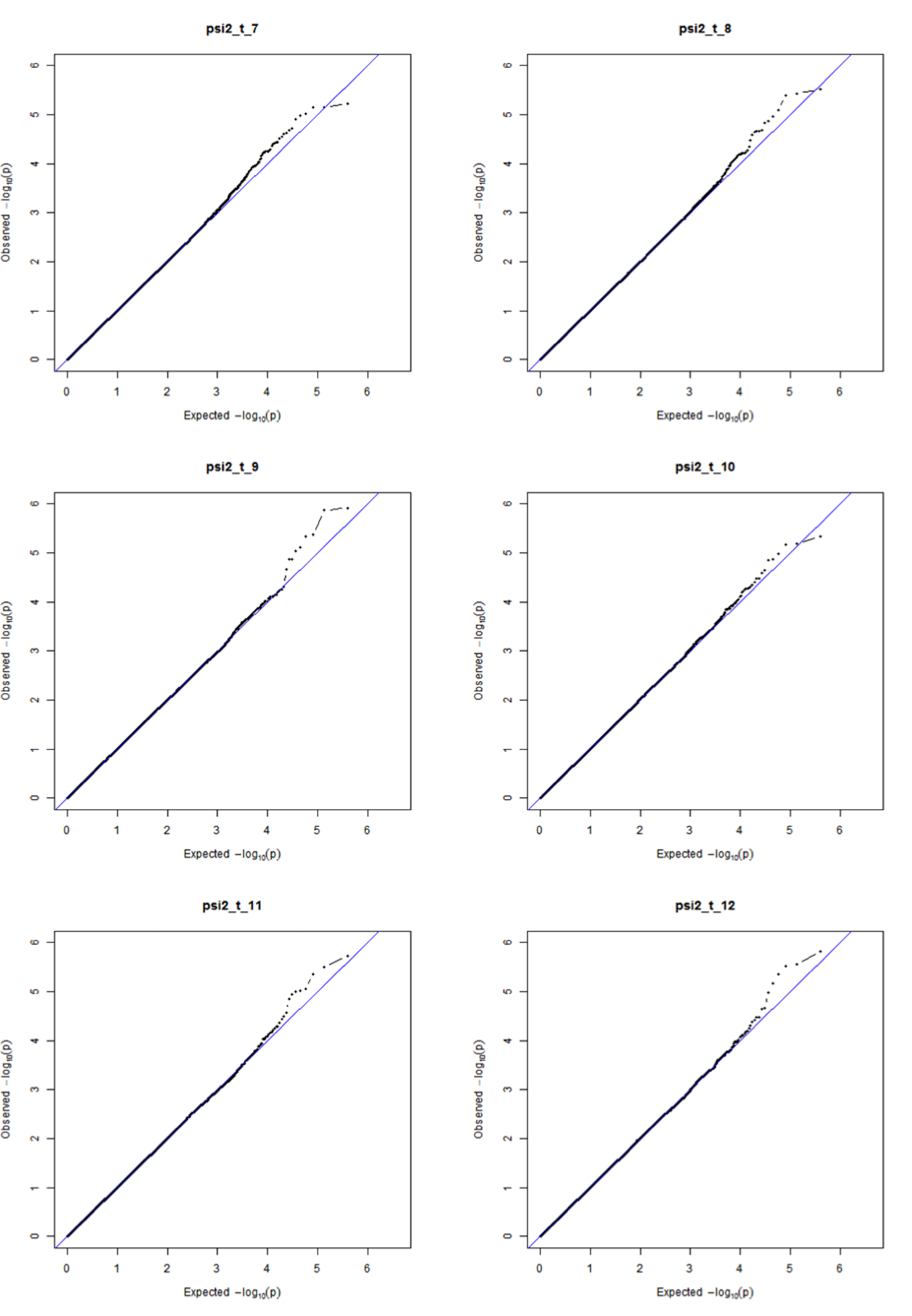

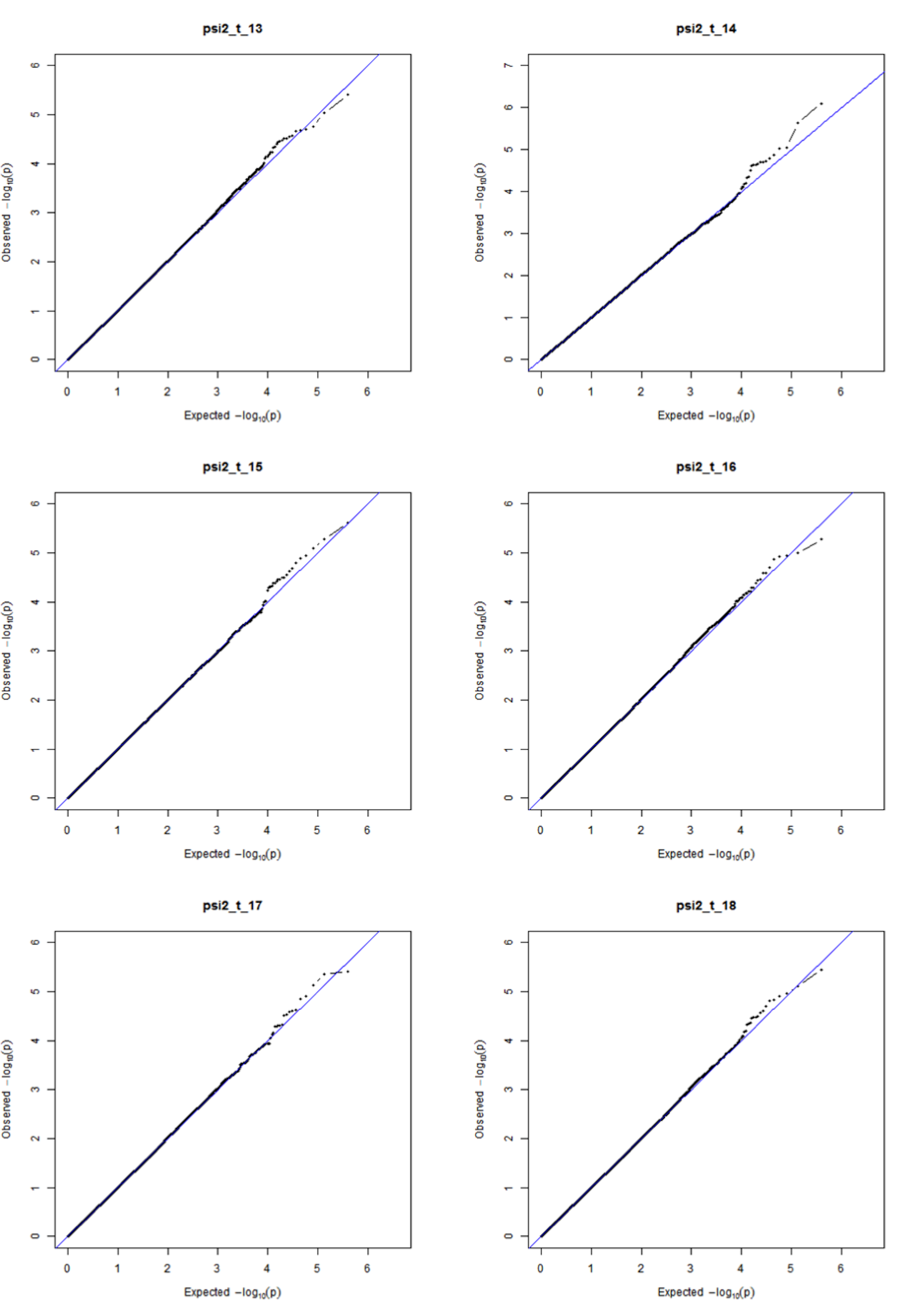
Supplementary Figure 8. Quantile-Quantile plots of the 18 time points used in this study.**

A test of association based on logistic regression for all SNPs of the GWAS, comparing Observed association scores (-log_10_(p)) with Expected association scores. The 18 plots represent the 18 time points used in this study in chronological order.

**Supplementary Table 1. Pearson correlation coefficients of Ф_PSII_ measurements.**

Different time points are compared indicated as “day (time point)”. All correlations are significant at p≤0.001. The dotted lines represents the switch from low light (100 μmol m^-2^ s^-1^) to high light (550 μmol m^-2^ s^-1^) irradiance at the onset of day 25.

| Days after sowing (time.point) | 23 (09.00h) | 23 (11.30h) | 23 (14.30h) | 24 (09.00h) | 24 (11.30h) | 24 (14.30h) | 25 (09.00h) | 25 (11.30h) | 25 (14.30h) | 26 (09.00h) | 26 (11.30h) | 26 (14.30h) | 27 (09.00h) | 27 (11.30h) | 27 (14.30h) | 28 (09.00h) | 28 (11.30h) |
| --- | --- | --- | --- | --- | --- | --- | --- | --- | --- | --- | --- | --- | --- | --- | --- | --- | --- |
| 23 (09.00h) |  |  |  |  |  |  |  |  |  |  |  |  |  |  |  |  |  |
| 23 (11.30h) | 0.91 |  |  |  |  |  |  |  |  |  |  |  |  |  |  |  |  |
| 23 (14.30h) | 0.88 | 0.91 |  |  |  |  |  |  |  |  |  |  |  |  |  |  |  |
| 24 (09.00h) | 0.87 | 0.87 | 0.84 |  |  |  |  |  |  |  |  |  |  |  |  |  |  |
| 24 (11.30h) | 0.87 | 0.89 | 0.85 | 0.90 |  |  |  |  |  |  |  |  |  |  |  |  |  |
| 24 (14.30h) | 0.86 | 0.87 | 0.89 | 0.89 | 0.89 |  |  |  |  |  |  |  |  |  |  |  |  |
| 25 (09.00h) | 0.33 | 0.32 | 0.35 | 0.38 | 0.32 | 0.38 |  |  |  |  |  |  |  |  |  |  |  |
| 25 (11.30h) | 0.31 | 0.29 | 0.34 | 0.35 | 0.29 | 0.37 | 0.94 |  |  |  |  |  |  |  |  |  |  |
| 25 (14.30h) | 0.26 | 0.26 | 0.30 | 0.29 | 0.26 | 0.31 | 0.89 | 0.95 |  |  |  |  |  |  |  |  |  |
| 26 (09.00h) | 0.25 | 0.24 | 0.30 | 0.29 | 0.24 | 0.30 | 0.87 | 0.90 | 0.92 |  |  |  |  |  |  |  |  |
| 26 (11.30h) | 0.29 | 0.28 | 0.32 | 0.34 | 0.29 | 0.35 | 0.87 | 0.90 | 0.91 | 0.96 |  |  |  |  |  |  |  |
| 26 (14.30h) | 0.29 | 0.28 | 0.31 | 0.34 | 0.29 | 0.34 | 0.85 | 0.89 | 0.89 | 0.94 | 0.98 |  |  |  |  |  |  |
| 27 (09.00h) | 0.23 | 0.20 | 0.25 | 0.28 | 0.23 | 0.28 | 0.85 | 0.87 | 0.86 | 0.94 | 0.94 | 0.93 |  |  |  |  |  |
| 27 (11.30h) | 0.31 | 0.28 | 0.31 | 0.36 | 0.32 | 0.37 | 0.83 | 0.84 | 0.82 | 0.88 | 0.93 | 0.95 | 0.96 |  |  |  |  |
| 27 (14.30h) | 0.32 | 0.29 | 0.32 | 0.37 | 0.33 | 0.37 | 0.81 | 0.82 | 0.79 | 0.85 | 0.90 | 0.93 | 0.94 | 0.99 |  |  |  |
| 28 (09.00h) | 0.25 | 0.20 | 0.24 | 0.30 | 0.26 | 0.30 | 0.81 | 0.81 | 0.79 | 0.84 | 0.85 | 0.87 | 0.95 | 0.95 | 0.95 |  |  |
| 28 (11.30h) | 0.33 | 0.29 | 0.31 | 0.38 | 0.34 | 0.39 | 0.76 | 0.76 | 0.71 | 0.76 | 0.81 | 0.85 | 0.88 | 0.95 | 0.96 | 0.95 |  |
| 28 (14.30h) | 0.32 | 0.28 | 0.31 | 0.37 | 0.34 | 0.38 | 0.73 | 0.73 | 0.68 | 0.73 | 0.78 | 0.82 | 0.86 | 0.93 | 0.96 | 0.94 | 0.98 |

**Supplementary Table 2. Broad sense heritability (H_2_) and marker based heritability (h_2_) per time point with their confidence intervals (left and right)**

| **Days after sowing (time.point)** | **H_2_** | **H_2_ conf.int.left** | **H_2_ conf.int.right** | **h_2_** | **h_2_ conf.int.left** | **h_2_ conf.int.right** |
| --- | --- | --- | --- | --- | --- | --- |
|
| 23 (09.00h) | 0.083 | 0.027 | 0.145 | 0.021 | 0.000 | 0.079 |
| 23 (11.30h) | 0.068 | 0.012 | 0.129 | 0.030 | 0.000 | 0.097 |
| 23 (14.30h) | 0.065 | 0.010 | 0.126 | 0.029 | 0.000 | 0.091 |
| 24 (09.00h) | 0.082 | 0.026 | 0.143 | 0.027 | 0.000 | 0.088 |
| 24 (11.30h) | 0.066 | 0.010 | 0.126 | 0.029 | 0.000 | 0.099 |
| 24 (14.30h) | 0.086 | 0.030 | 0.148 | 0.015 | 0.000 | 0.068 |
| 25 (09.00h) | 0.198 | 0.137 | 0.263 | 0.304 | 0.013 | 0.596 |
| 25 (11.30h) | 0.228 | 0.166 | 0.292 | 0.392 | 0.050 | 0.734 |
| 25 (14.30h) | 0.213 | 0.151 | 0.278 | 0.297 | 0.025 | 0.570 |
| 26 (09.00h) | 0.263 | 0.201 | 0.328 | 0.355 | 0.023 | 0.686 |
| 26 (11.30h) | 0.321 | 0.259 | 0.385 | 0.521 | 0.114 | 0.928 |
| 26 (14.30h) | 0.335 | 0.273 | 0.399 | 0.489 | 0.103 | 0.875 |
| 27 (09.00h) | 0.267 | 0.205 | 0.332 | 0.403 | 0.036 | 0.769 |
| 27 (11.30h) | 0.310 | 0.248 | 0.374 | 0.426 | 0.054 | 0.797 |
| 27 (14.30h) | 0.310 | 0.247 | 0.374 | 0.423 | 0.061 | 0.785 |
| 28 (09.00h) | 0.266 | 0.204 | 0.331 | 0.369 | 0.023 | 0.716 |
| 28 (11.30h) | 0.274 | 0.212 | 0.339 | 0.384 | 0.025 | 0.742 |
| 28 (14.30h) | 0.296 | 0.233 | 0.360 | 0.325 | 0.007 | 0.644 |

**Supplementary Table 3. List of 173 re-sequenced Arabidopsis accessions used for haplotype analysis.**

‘Code’ represents the maintainer code from the Arabidopsis Biological Resource Center ([https://abrc.osu.edu](https://abrc.osu.edu/)), and ‘Accession’ represents the commonly accepted abbreviated name for the Arabidopsis accession (http://www.arabidopsis.org/abrc/catalog/natural_accession_1.html).

| Code | Accession | Code | Accession | Code | Accession | Code | Accession |
| --- | --- | --- | --- | --- | --- | --- | --- |
| cs22689 | RRS-10 | cs28759 | Ting-1 | cs76146 | HSm | cs76232 | Ste-3 |
| cs28013 | Alst-1 | cs28779 | Tscha-1 | cs76147 | In-0 | cs76235 | T1080 |
| cs28014 | Amel-1 | cs28780 | Tsu-0 | cs76148 | JEA | cs76236 | T1110 |
| cs28018 | Ang-0 | cs28786 | Ty-0 | cs76150 | Kas-1 | cs76237 | T1130 |
| cs28049 | Ann-1 | cs28787 | Uk-1 | cs76152 | Kelsterbach-4 | cs76239 | T540 |
| cs28053 | Ba-1 | cs28795 | Utrecht | cs76153 | Kin-0 | cs76242 | Ta-0 |
| cs28054 | Baa-1 | cs28800 | Ven-1 | cs76154 | Kno-18 | cs76244 | Tamm-2 |
| cs28064 | Benk-1 | cs28804 | Wa-1 | cs76156 | Kulturen-1 | cs76245 | TDr-1 |
| cs28091 | Boot-1 | cs28822 | Wl-0 | cs76159 | Lc-0 | cs76246 | TDr-17 |
| cs28099 | Bsch-0 | cs76087 | Ag-0 | cs76164 | Ler-1 | cs76249 | TDr-8 |
| cs28128 | Ca-0 | cs76088 | Alc-0 | cs76166 | Liarum | cs76250 | Tomegap-2 |
| cs28135 | Chat-1 | cs76091 | An-1 | cs76167 | Lilloe-1 | cs76251 | Tottarp-2 |
| cs28142 | CIBC-5 | cs76092 | App1-16 | cs76168 | Lip-0 | cs76268 | Ts-1 |
| cs28160 | Cnt-1 | cs76093 | Baa1-2 | cs76170 | Lis-2 | cs76293 | Ull2-3 |
| cs28193 | Com-1 | cs76094 | Bay-0 | cs76171 | Lisse | cs76294 | Ull2-5 |
| cs28201 | Da(1)-12 | cs76096 | Bg-2 | cs76172 | LL-0 | cs76296 | Uod-7 |
| cs28210 | Do-0 | cs76097 | Bla-1 | cs76173 | Lm-2 | cs76297 | Van-0 |
| cs28241 | Es-0 | cs76098 | Blh-1 | cs76174 | Lom1-1 | cs76298 | Vaar2-1 |
| cs28279 | Gel-1 | cs76099 | Bor-1 | cs76175 | Lov-5 | cs76301 | Wei-0 |
| cs28280 | Gie-0 | cs76100 | Bor-4 | cs76176 | Lp2-2 | cs76302 | Wil-1 |
| cs28336 | Ha-0 | cs76101 | Br-0 | cs76177 | Lp2-6 | cs76303 | Ws-0 |
| cs28343 | Hau-0 | cs76102 | Broet1-6 | cs76178 | Lund | cs76304 | Wt-5 |
| cs28344 | Hey-1 | cs76103 | Bu-0 | cs76191 | Mrk-0 | cs76305 | Yo-0 |
| cs28345 | Hh-0 | cs76105 | Bur-0 | cs76192 | Mt-0 |  |  |
| cs28350 | Hn-0 | cs76106 | C24 | cs76193 | Mz-0 |  |  |
| cs28364 | Je-0 | cs76109 | Can-0 | cs76194 | N13 |  |  |
| cs28369 | Jl-3 | cs76111 | CIBC-17 | cs76195 | Na-1 |  |  |
| cs28394 | Kl-5 | cs76113 | Col-0 | cs76196 | NC-6 |  |  |
| cs28395 | Kn-0 | cs76114 | Ct-1 | cs76198 | NFA-10 |  |  |
| cs28420 | Kro-0 | cs76116 | Cvi-0 | cs76199 | NFA-8 |  |  |
| cs28490 | Mc-0 | cs76117 | Dra3-1 | cs76200 | oemoe2-1 |  |  |
| cs28492 | Mh-0 | cs76118 | DraII-1 | cs76203 | Oy-0 |  |  |
| cs28495 | Mnz-0 | cs76124 | Duk | cs76210 | Per-1 |  |  |
| cs28527 | Nc-1 | cs76125 | Eden-2 | cs76212 | PHW-34 |  |  |
| cs28564 | No-0 | cs76126 | Edi-0 | cs76213 | Pna-17 |  |  |
| cs28573 | Nw-0 | cs76127 | Est-1 | cs76214 | Pro-0 |  |  |
| cs28578 | Nz1 | cs76128 | FÃeb-4 | cs76215 | Pu2-23 |  |  |
| cs28583 | Old-1 | cs76129 | Fei-0 | cs76216 | Ra-0 |  |  |
| cs28587 | Or-0 | cs76131 | FjÃe1-2 | cs76217 | Rak-2 |  |  |
| cs28640 | Pla-0 | cs76132 | FjÃe1-5 | cs76218 | Ren-1 |  |  |
| cs28650 | Pog-0 | cs76133 | Ga-0 | cs76219 | Rev-2 |  |  |
| cs28685 | Rhen-1 | cs76135 | Ge-0 | cs76220 | Rmx-A180 |  |  |
| cs28692 | Rou-0 | cs76136 | Got-7 | cs76222 | Rsch-4 |  |  |
| cs28713 | RRS-7 | cs76137 | Gr-1 | cs76223 | Sanna-2 |  |  |
| cs28725 | Sav-0 | cs76139 | Gy-0 | cs76224 | Sap-0 |  |  |
| cs28729 | Sei-0 | cs76140 | Hi-0 | cs76226 | Se-0 |  |  |
| cs28732 | Sg-1 | cs76141 | Hod | cs76227 | Shahdara |  |  |
| cs28739 | Si-0 | cs76142 | Hov4-1 | cs76229 | Sparta-1 |  |  |
| cs28743 | Sp-0 | cs76143 | Hovdala-2 | cs76230 | Sq-8 |  |  |
| cs28758 | Tha-1 | cs76145 | Hs-0 | cs76231 | St-0 |  |  |

**Supplementary Table 4. Primer sequences**

Sequences (5’-3’) of oligonucleotide primers used for real-time quantitative Reverse Transcriptase-PCR of candidate and reference genes.

| Gene | Forward Primer | Reverse Primer |
| --- | --- | --- |
| *YS1* | GCCTCGCGTAACCACAAATC | TTTACGCCGAGTGTGGAGAG |
| *DGS1* | GAGTGGGAAGCAAGCAGTCA | GAGTTAGGAAGGCCACAGCA |
| *TrnD* | GGGATTGTAGTTCAATTGGTCAGAGC | CGGGACTGACGGGGCTCGAAC |
| *TrnW* | ACGCTCTTAGTTCAGTTCGGTAG | CACGCTCTGTAGGATTTGAACC |
| *UBQ7* | GCAGCGACACCATCGACAAT | AGGTCCGGCCATCTTCCAAT |
| *CB5E* | TGATCATCCTGGAGGCGATG | TTGCAGTGTCGCTGTGACCA |
