# Supplementary material for: Natural variation of YELLOW SEEDLING1 affects photosynthetic acclimation of Arabidopsis thaliana
Source: Nat Commun. 2017 Nov 10;8:1421. doi: 10.1038/s41467-017-01576-3 (PMC5680337; doi:10.1038/s41467-017-01576-3)
Supplement: Supplementary file 3 — Descriptions of Additional Supplementary Files [file 41467_2017_1576_MOESM3_ESM.pdf]

Descriptions of Additional Supplementary Files

File Name: Supplementary Dataset 1
Descriptions: List of candidate genes co-localized with SNPs repetitively associated with ΦPSII with an LD window of 100Kb

File Name: Supplementary Dataset 2
Descriptions: Shortlist of prioritized candidate genes potentially underlying quantitative trait loci (QTLs) for photosynthesis acclimation in Arabidopsis.

File Name: Supplementary Dataset 3
Descriptions: List of candidate genes co-localized with SNPs repetitively associated with ΦPSII with an LD window of 20Kb

File Name: Supplementary Dataset 4
Descriptions: List of candidate genes co-localized with SNPs associated with curve parameters of ΦPSII response

File Name: Supplementary Dataset 5
Descriptions: List of candidate genes co-localized with SNPs associated with first principal component of ΦPSII response
